# An integrative description of *Macrobiotus shonaicus* sp. nov. (Tardigrada: Macrobiotidae) from Japan with notes on its phylogenetic position within the *hufelandi* group

**DOI:** 10.1371/journal.pone.0192210

**Published:** 2018-02-28

**Authors:** Daniel Stec, Kazuharu Arakawa, Łukasz Michalczyk

**Affiliations:** 1 Institute of Zoology and Biomedical Research, Jagiellonian University, Gronostajowa 9, Kraków, Poland; 2 Institute for Advanced Biosciences, Keio University, Mizukami 246–2, Kakuganji, Tsuruoka, Yamagata, Japan; University of Ostrava, CZECH REPUBLIC

## Abstract

Tardigrade research in Japan dates back over 100 years, and to date, 167 species of this ecdysozoan phylum have been reported from the country. Of these species, the *Macrobiotus hufelandi* complex has been represented only by the nominal taxon of this group, *Macrobiotus hufelandi*. In this article, a new species of the *hufelandi* group from Japan, *Macrobiotus shonaicus* **sp. nov.**, is described using integrative taxonomy. In addition to the detailed morphological and morphometric data, obtained using phase contrast light microscopy (PCM) and scanning electron microscopy (SEM), we provide DNA sequences of four molecular markers (both nuclear and mitochondrial). The new species belongs to the *persimilis* subgroup and is most similar to *M*. *anemone* from USA, *M*. *naskreckii* from Mozambique, and *M*. *patagonicus* from Argentina, but it can be easily distinguished from these species by the presence of thin flexible filaments on terminal discs of the egg process. By the latter character, the new species is most similar to *M*. *paulinae* and *M*. *polypiformis*, but it can be easily distinguished from them by having a solid egg surface between egg processes (*i*.*e*., without pores or reticulum). A phylogenetic analysis of available DNA sequences of the COI marker for the *hufelandi* group revealed that the new species clusters with the two other species that exhibit filaments on egg process discs (*M*. *paulinae* and *M*. *polypiformis*) and with two species that have entire egg processes modified into filaments (*M*. *kristenseni* and *M*. *scoticus*). All five species form a clade distinct from all other sequenced species of the *hufelandi* group with typical mushroom- or inverted goblet-shaped egg processes, which may suggest that the ancestor of the five species with atypical egg processes had a mutation allowing derivations from the mushroom or inverted chalice-like shape of egg processes.

## Introduction

Terrestrial tardigrades are micrometazoans most commonly found in mosses, lichens, leaf litter and soil [1]. The phylogenetic position of the phylum Tardigrada is still uncertain, with some studies placing this phylum as a sister group of Arthropoda and Onychophora within the megaclade Panarthropoda, and some others placing tardigrades as a sister group to Nematoda [2 and the literature cited therein]. Until now, over 1200 species have been known to reside within this phylum [3], and approximately twenty new species are described each year [4].

The first report on tardigrades from Japan come from a zoology textbook by Iijima [5]. Currently, Japanese tardigrade fauna include 167 species, of which 26 were originally described from Japan [6]. The family Macrobiotidae is represented by 16 species, but some records—such as *Mesobiotus harmsworthi* (Murray, 1907) [7], *Minibiotus intermedius* (Plate, 1888) [8], *Paramacrobiotus areolatus* (Murray, 1907) [7], *Paramacrobiotus richtersi* (Murray, 1911) [9] and *Macrobiotus hufelandi* C.A.S. Schultze, 1834 [10]—should be treated with great caution since they are nominal taxa for species complexes for which the original descriptions are incomplete and outdated, making the exact identification almost impossible. The globally distributed *Macrobiotus hufelandi* complex has been represented until now only by the nominal taxon of this group, *M*. *hufelandi*, and no other *hufelandi* species has been reported from Japan to date [6].

In this article, we describe a new tardigrade species of the *hufelandi* group, *Macrobiotus shonaicus* **sp. nov.**, from Japan. The integrative description and species delineation involved morphological and morphometric data obtained using phase contrast light microscopy (PCM) and scanning electron microscopy (SEM) as well as molecular data in the form of DNA sequences for four molecular markers (nuclear: 18S rRNA, 28S rRNA, ITS-2, and mitochondrial COI). The new species belongs to the *persimilis* subgroup (with a solid egg surface between processes), but it has modified egg processes with some teeth of terminal discs elongated to flexible filaments, such as those found in two other recently described species: *Macrobiotus paulinae* Stec, Smolak, Kaczmarek & Michalczyk, 2015 [11] from Africa and *Macrobiotus polypiformis* Roszkowska, Ostrowska, Stec, Janko & Kaczmarek, 2017 [12] from South America.

## Materials and methods

### Sample processing and tardigrade culturing

A sample of moss *Bryum argenteum* growing on a car park’s concrete surface containing the new species was collected from Otsuka-machi, Tsuruoka-City, Japan (38°44’24”N, 139°48’26”E; 13 m asl) in May 2016 by KA. The place of collection was a parking lot of an apartment KA rented, and specific permission was not required. The authors confirm that our sampling did not involve endangered or protected species. The sample was collected and examined for terrestrial tardigrades using a stereo microscope SZ61 (Olympus). Ten individuals of the new species were extracted from the sample and placed in an *in vitro* culture in five separate pairs. Only one of the five cultures successfully proliferated. Specimens of this isogenic strain were reared using a previously described protocol for *Hypsibius dujardini* (Doyère, 1840) [13, 14]. Briefly, tardigrades were fed *Chlorella vulgaris* (Chlorella Industry) on 2% Bacto Agar (Difco) plates prepared with Volvic water, and the plates were incubated at 18°C under constant darkness. Culture plates were renewed every 7–8 days.

### Microscopy and imaging

Specimens for light microscopy were mounted on microscope slides in a small drop of Hoyer’s medium and secured with a cover slip, following the protocol established by Morek et al. [15]. Slides were then dried for five days at 60°C. Dried slides were sealed with a transparent nail polish and examined under a *Nikon Eclipse 50i* phase contrast light microscope (PCM) associated with a *Nikon Digital Sight DS-L2* digital camera. To obtain clean and extended specimens for SEM, tardigrades were processed according to the protocol established by Stec et al. [11]. In short, specimens were first subjected to a 60°C water bath for 30 min to obtain fully extended animals, followed by a water/ethanol and an ethanol/acetone series, and then followed by CO_2_ critical point drying. Specimens were finally sputter coated with a thin layer of gold. Specimens were examined under high vacuum in a *Versa 3D DualBeam* Scanning Electron Microscope at the ATOMIN facility of Jagiellonian University, Kraków, Poland. The type population was also examined for the presence of males using aceto-orcein staining following Stec et al. [16].

All figures were assembled in *Corel Photo-Paint X6*, ver. 16.4.1.1281. For deep structures that could not be fully focused in a single photograph, a series of 2–8 images were taken every *ca*. 0.2 μm and then assembled manually into a single deep-focus image.

### Morphometrics and morphological nomenclature

All measurements are given in micrometers (μm). Sample size was adjusted following recommendations by Stec et al. [17]. Structures were measured only if their orientation was suitable. Body length was measured from the anterior extremity to the end of the body, excluding the hind legs. The terminology used to describe oral cavity armature (OCA) follows Michalczyk & Kaczmarek [18]. Buccal tube length and the level of the stylet support insertion point were measured according to Pilato [19]. Buccal tube width was measured as the external and internal diameters at the level of the stylet support insertion point. Macroplacoid length sequence is given according to Kaczmarek et al. [20]. The lengths of the claw branches were measured from the base of the claw (*i*.*e*., excluding the lunula) to the top of the branch, including accessory points [21]. The *pt* index is the ratio of the length of a given structure to the length of the buccal tube expressed as a percentage [19]. The distance between egg processes was measured as the shortest line connecting the base edges of the two closest processes [21]. Morphometric data were handled using the “Parachela” ver. 1.2 template available from the Tardigrada Register [4]. Tardigrade taxonomy follows Bertolani et al. [22].

### Comparative material

The taxonomic key for the *hufelandi* group by Kaczmarek & Michalczyk [21] was used to determine whether the isolated species had already been described. After the species could not be identified with the key, we compared it with the original descriptions of the most similar *hufelandi* group species, which have solid egg surfaces (*persimilis* group, 11 species): *M*. *anemone* Meyer, Domingue & Hinton, 2014 [23], *M*. *halophilus* Fontoura, Rubal & Veiga, 2017 [24], *M*. *hyperboreus* Biserov, 1990 [25], *M*. *kazmierskii* Kaczmarek & Michalczyk, 2009 [26], *M*. *kristenseni* Guidetti, Peluffo, Rocha, Cesari & Moly de Peluffo, 2013 [27], *M*. *marlenae* Kaczmarek & Michalczyk, 2004 [28], *M*. *naskreckii* Bąkowski, Roszkowska, Gawlak & Kaczmarek, 2016 [29], *M*. *patagonicus* Maucci, 1988 [30], *M*. *persimilis* Binda & Pilato, 1972 [31], *M*. *polonicus* Pilato, Kaczmarek, Michalczyk & Lisi, 2003 [32], and *M*. *recens* Cuénot, 1932 [33]. Moreover, two species that like the new species exhibit flexible filaments at the terminal disc edge of the egg process were used as comparative material in this study: *M*. *paulinae* and *M*. *polypiformis*.

### Genotyping

DNA was extracted from individual animals following a *Chelex*^*®*^ *100* resin (*Bio-Rad*) extraction method established by Casquet et al. [34] with modifications described in detail in Stec et al. [11]. We sequenced four DNA fragments differing in mutation rates (from the most to least conservative): the small ribosome subunit (18S rRNA, nDNA), the large ribosome subunit (28S rRNA, nDNA), the internal transcribed spacer (ITS-2, nDNA), and the cytochrome oxidase subunit I (COI, mtDNA). All fragments were amplified and sequenced according to the protocols described in Stec et al. [11]; primers and original references for specific PCR programs are listed in Table 1. Sequencing products were read with the *ABI 3130xl* sequencer at the Molecular Ecology Lab, Institute of Environmental Sciences of the Jagiellonian University, Kraków, Poland. Sequences were processed in *BioEdit* ver. 7.2.5 [35] and submitted to GenBank.

**Table 1:** Primers and references for PCR protocols for amplification of the four DNA fragments sequenced in the study.

DNA fragment	Primer name	Primer direction	Primer sequence (5’-3’)	Primer source	PCR program
**18S rRNA**	18S_Tar_Ff1	forward	AGGCGAAACCGCGAATGGCTC	Stec et al. [36]	Zeller [37]
	18S_Tar_Rr1	reverse	GCCGCAGGCTCCACTCCTGG		
**28S rRNA**	28SF0001	forward	ACCCVCYNAATTTAAGCATAT	Mironov et al. [38]	Mironov et al. [38]
	28SR0990	reverse	CCTTGGTCCGTGTTTCAAGAC		
**ITS-2**	ITS2_Eutar_Ff	forward	CGTAACGTGAATTGCAGGAC	Stec et al. [41]	Stec et al. [41]
	ITS2_Eutar_Rr	reverse	TCCTCCGCTTATTGATATGC		
**COI**	LCO1490	forward	GGTCAACAAATCATAAAGATATTGG	Folmer et al. [39]	Michalczyk et al. [40]
	HCO2198	reverse	TAAACTTCAGGGTGACCAAAAAATCA		

### Comparative analysis

For molecular comparisons, all the published sequences of the four abovementioned markers for species of the *hufelandi* group were downloaded from GenBank (listed in Table 2). The sequences were aligned using the default settings in MAFFT version 7 [42, 43] and manually checked against non-conservative alignments in *BioEdit* ver. 7.2.5 [35]. Then, the aligned sequences were trimmed to 773 (18S rRNA), 711 (28S rRNA), 307 (ITS-2), and 621 (COI), bp. All COI sequences were translated into protein sequences in MEGA7 [44] to check against pseudogenes. Uncorrected pairwise distances were calculated using MEGA version 7.0 [44]. Despite the fact that genetic distances in barcoding studies are frequently calculated in accordance with the Kimura 2 parameter model, as proposed by Hebert et al. [45], the more recent work by Srivathsan & Meier [46] showed that this model of nucleotide evolution is poorly justified. Moreover, Srivathsan & Meier [46] showed that uncorrected p-distances may provide a comparable or even a higher success rate of taxon delimitation than distances computed under the K2P. Therefore, we used basic p-distances in all our analyses.

**Table 2:** Sequences used for molecular comparisons and phylogenetic analyses of *Macrobiotus shonaicus* sp. nov. with all other species of the *Macrobiotus hufelandi* group for which DNA sequences are currently available.

DNA Marker	Species	Accession number	Source
**18S**	*M*. *hufelandi* C.A.S. Schultze, 1834 [10]	GQ849024	Giribet et al. [63],
	*M*. *hufelandi* gr	HQ604971, FJ435738–40	Bertolani et al. [22], Guil & Giribet [62]
	*M*. *joannae* Pilato & Binda, 1983 [64]	HQ604974–5	Bertolani et al. [22]
	*M*. *kristenseni* Guidetti et al., 2013 [27]	KC193577	Guidetti et al. [27]
	*M*. *macrocalix* Bertolani & Rebecchi, 1993 [54]	HQ604976	Bertolani et al. [22]
	*M*. *paulinae* Stec et al., 2015 [11]	KT935502	Stec et al. [11]
	*M*. *polypiformis* Roszkowska et al., 2017 [12]	KX810008	Roszkowska et al. [12]
	*M*. *polonicus* Pilato et al., 2003 [32]	HM187580	Wełnicz et al. [49]
	*M*. *sapiens* Binda & Pilato, 1984 [50]	DQ839601	Bertolani et al. [22]
	*M*. *scoticus* Stec et al., 2017 [52]	KY797265	Stec et al. [52]
**28S**	*M*. *hufelandi* gr	FJ435751, FJ435754–5	Guil & Giribet [62]
	*M*. *paulinae* Stec et al., 2015 [11]	KT935501	Stec et al. [11]
	*M*. *polypiformis* Roszkowska et al., 2017 [12]	KX810009	Roszkowska et al. [12]
	*M*. *scoticus* Stec et al., 2017 [52]	KY797266	Stec et al. [52]
**ITS-2**	*M*. *paulinae* Stec et al., 2015 [11]	KT935500	Stec et al. [11]
	*M*. *polonicus* Pilato et al., 2003 [32]	HM150647	Wełnicz et al. [49]
	*M*. *polypiformis* Roszkowska et al., 2017 [12]	KX810010	Roszkowska et al. [12]
	*M*. *sapiens* Binda & Pilato, 1984 [50]	GQ403680	Schill et al. [51]
	*M*. *scoticus* Stec et al., 2017 [52]	KY797268	Stec et al. [52]
**COI**	*M*.cf. *hufelandi*	HQ876589–94, HQ876596	Bertolani et al. [53]
	*M*. *h*. *hufelandi* C.A.S. Schultze, 1834 [10]	HQ876584, HQ876586–8	Bertolani et al. [53]
	*M*. *kristenseni* Guidetti et al., 2013 [27]	KC193575–6	Guidetti et al. [27]
	*M*. *macrocalix* Bertolani & Rebecchi, 1993 [54]	FJ176203–17, HQ876571	Cesari et al. [55], Bertolani et al. [53]
	*M*. *paulinae* Stec et al., 2015 [11]	KT951668	Stec et al. [11]
	*M*. *polypiformis* Roszkowska et al., 2017 [12]	KX810011–2	Roszkowska et al. [12]
	*M*. *sandrae* Bertolani & Rebecchi, 1993 [54]	HQ876566-70, HQ876572–83	Bertolani et al. [53]
	*M*. *scoticus* Stec et al., 2017 [52]	KY797267	Stec et al. [52]
	*M*. *terminalis* Bertolani & Rebecchi, [54]	JN673960, AY598775	Cesari et al. [56], Guidetti et al. [57]
	*M*. *vladimiri* Bertolani et al., 2011 [58]	HM136931–4, HQ876568	Bertolani et al. [53, 58]
outgroup	*M*. cf. *alpigenum*	KU513422	Kosztyła et al. [59]
	*M*. *berladnicorum* Ciobanu et al., 2014 [60]	KT951659	Morek et al. [61]
	*M*. *variefidum* Morek et al., 2016 [61]	KT951663	Morek et al. [61]

### Phylogenetic analysis

To verify the phylogenetic position of the new species, a phylogenetic tree was constructed on published COI sequences of species from the *hufelandi* group with three *Milnesium* species as the outgroup (see Table 2 for references). Since the COI is a protein-coding gene, before partitioning, we divided our alignment into three data blocks constituting three separate codon positions using PartitionFinder version 2.1.1 [47] under the Bayesian Information Criterion (BIC). The best schemes for partitioning and substitution models were chosen for posterior phylogenetic analysis. First, we ran the analysis to test all possible models implemented in the program. As a best-fit partitioning scheme, PartitionFinder suggested retaining three predefined partitions separately. The best-fit models for these partitions were F81+I for the first codon position, TRN+G for the second codon position and SYM+I for the third codon position. Since RAxML [48] allows only a single model of rate heterogeneity (of the GTR family) in partitioned analyses, we additionally tested GTR, GTR+I, GTR+G and GTR+I+G using PartitionFinder. The best-fit model for all partitions in this analysis was GTR+G.

Maximum-likelihood (ML) topologies were constructed using RAxML v8.0.19 [48]. The strength of support for internal nodes of ML construction was measured using 1000 rapid bootstrap replicates. Bootstrap (BS) support values ≥70% on the final tree were regarded as significant statistical support. Bayesian inference (BI) marginal posterior probabilities were calculated using MrBayes v3.2 [65]. Random starting trees were used, and the analysis was run for eight million generations, sampling the Markov chain every 1000 generations. An average standard deviation of split frequencies of <0.01 was used as a guide to ensure the two independent analyses had converged. The program Tracer v1.3 [66] was then used to ensure Markov chains had reached stationarity and to determine the correct ‘burn-in’ for the analysis, which was the first 10% of generations. A consensus tree was obtained after summarizing the resulting topologies and discarding the ‘burn-in’. Based on the BI consensus tree, clades recovered with a posterior probability (PP) between 0.95 and 1 were considered well supported, those with a PP between 0.90 and 0.94 were considered moderately supported, and those with a lower PP were considered unsupported. All final consensus trees were viewed in and visualized by FigTree v.1.4.3, available from http://tree.bio.ed.ac.uk/software/figtree.

### Data deposition

Raw morphometric measurements underlying the description of *Macrobiotus shonaicus* **sp. nov.** are given in supplementary materials (S1 File) and are additionally deposited in the Tardigrada Register [4] under www.tardigrada.net/register/0051.htm. The DNA sequences for the type population are deposited in GenBank (https://www.ncbi.nlm.nih.gov/genbank). Uncorrected pairwise distances are given in the supplementary materials (S2 File).

### Nomenclatural acts

The electronic edition of this article conforms to the requirements of the amended International Code of Zoological Nomenclature, and hence the new names contained herein are available under that Code from the electronic edition of this article. This published work and the nomenclatural acts it contains have been registered in ZooBank, the online registration system for the ICZN. The ZooBank LSIDs (Life Science Identifiers) can be resolved and the associated information viewed through any standard web browser by appending the LSID to the prefix "http://zoobank.org/". The LSID for this publication is: urn:lsid:zoobank.org:pub:FE5D4FE7-11F9-48C9-B1E2-9EA516CCBD08. The electronic edition of this work was published in a journal with an ISSN, and has been archived and is available from the following digital repositories: PubMed Central, LOCKSS.

## Results

### Taxonomic account of the new species

**Phylum:** Tardigrada Doyère, 1840 [13]

**Class:** Eutardigrada Richters, 1926 [67]

**Order:** Parachela Schuster, Nelson, Grigarick & Christenberry, 1980 [68]

**Superfamily:** Macrobiotoidea Thulin, 1928 [69] (in Marley et al. [70])

**Family:** Macrobiotidae Thulin, 1928 [69]

**Genus:** *Macrobiotus* C.A.S. Schultze, 1834 [10]

**Fig 1:**
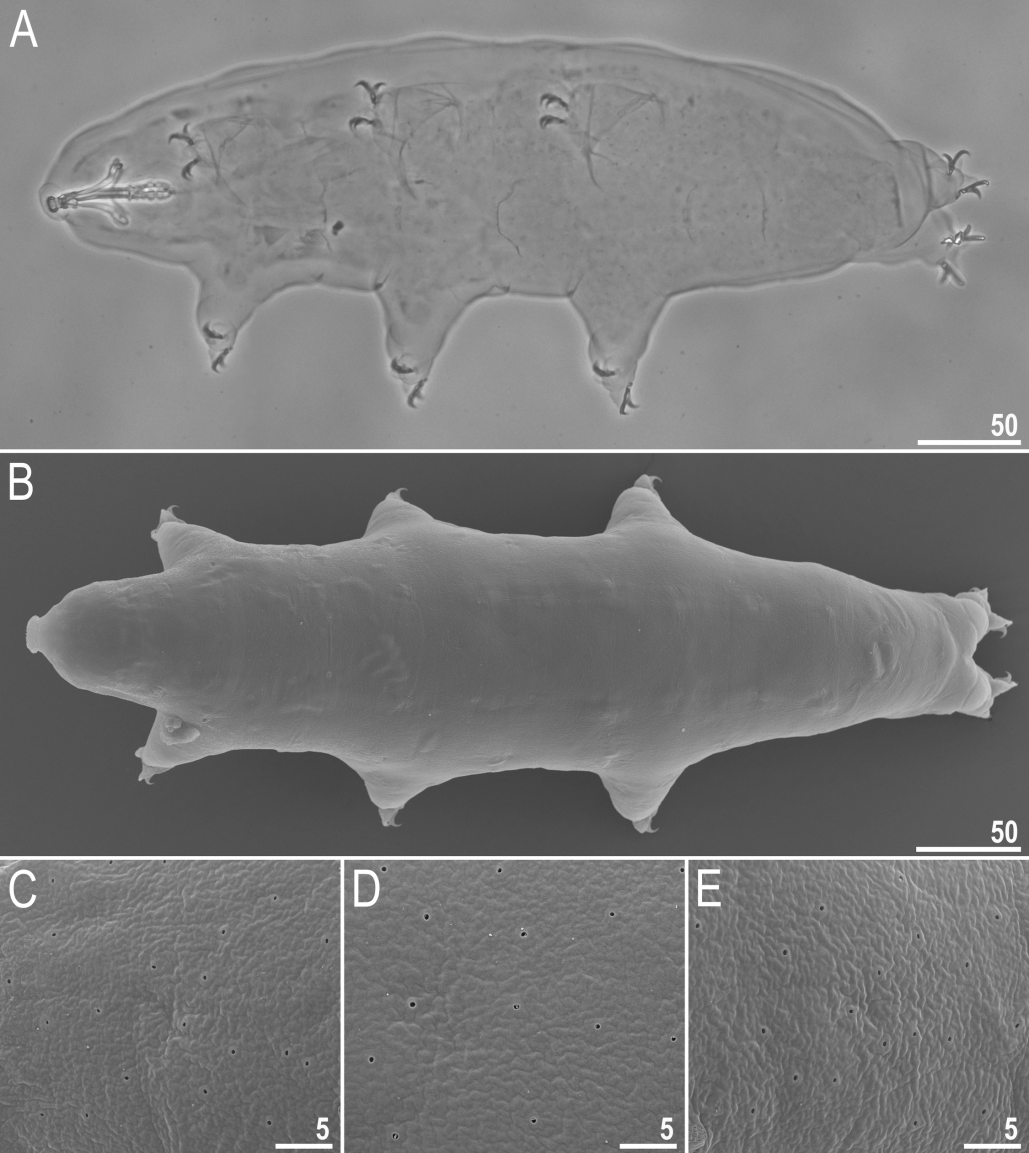
*Macrobiotus shonaicus* sp. nov.–habitus. **A**–dorso-ventral projection (holotype, Hoyer’s medium, PCM); **B**–dorsal view (paratype, SEM); and **C–E**–cuticular pores seen in SEM on the anterior (C), median (D) and posterior (E) part of the body (paratype). Scale bars in μm.

**Fig 2:**
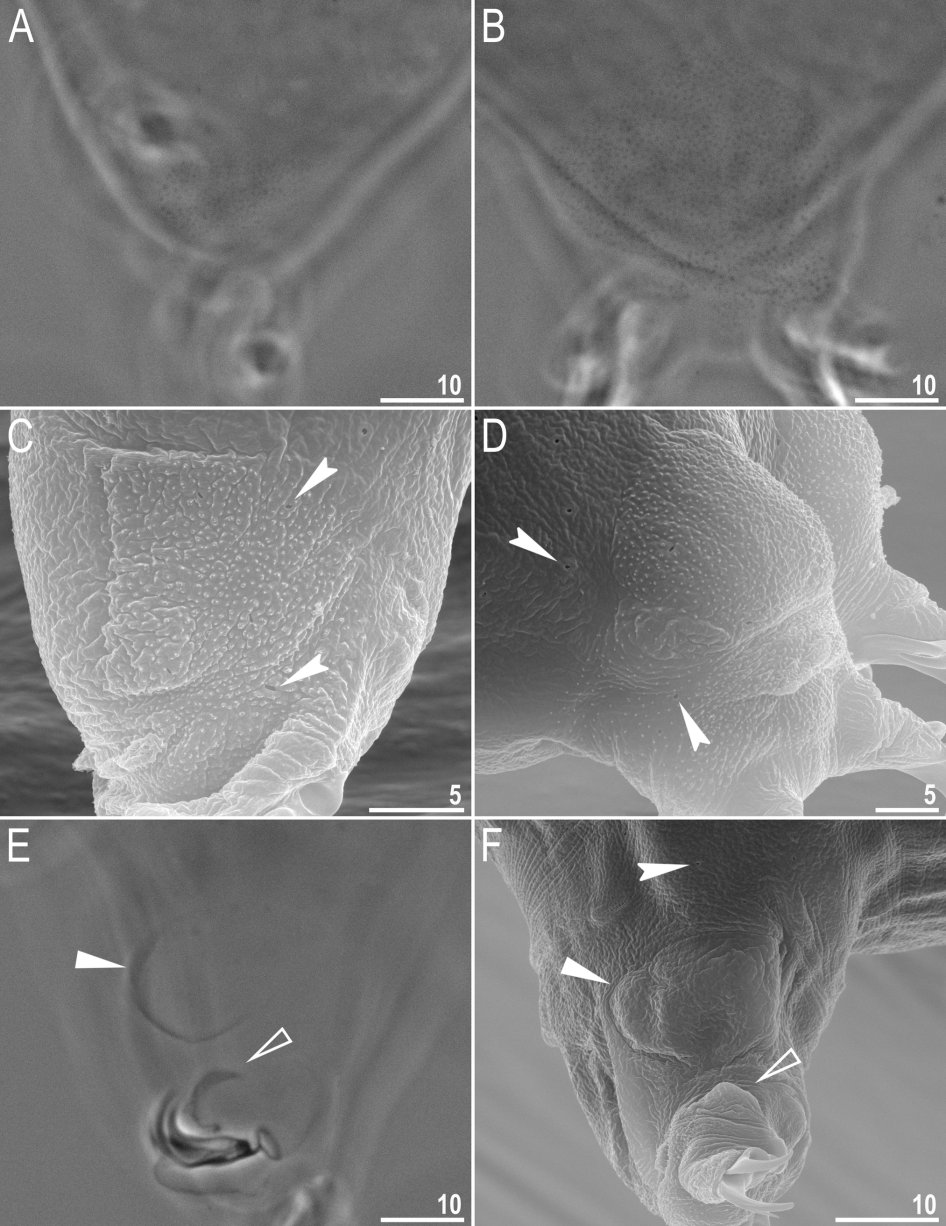
*Macrobiotus shonaicus* sp. nov.–cuticular structures on legs. **A–**granulation on leg II (paratype, PCM); **B–**granulation on leg IV (paratype, PCM); **C**–granulation on leg II (paratype, SEM); **D**–granulation on leg IV (SEM); **E–**cuticular bulge resembling pulvinus like structure and faint cuticular fold on the internal surface of leg II (holotype, PCM); **F**–cuticular bulge resembling pulvinus like structure and faint cuticular fold on the internal surface of leg III (paratype, SEM); Indented arrowheads indicate pores on legs, filled flat arrowheads indicate the cuticular bulge, whereas empty flat arrowheads indicate faint cuticular fold under the claws. Scale bars in μm.

**Fig 3:**
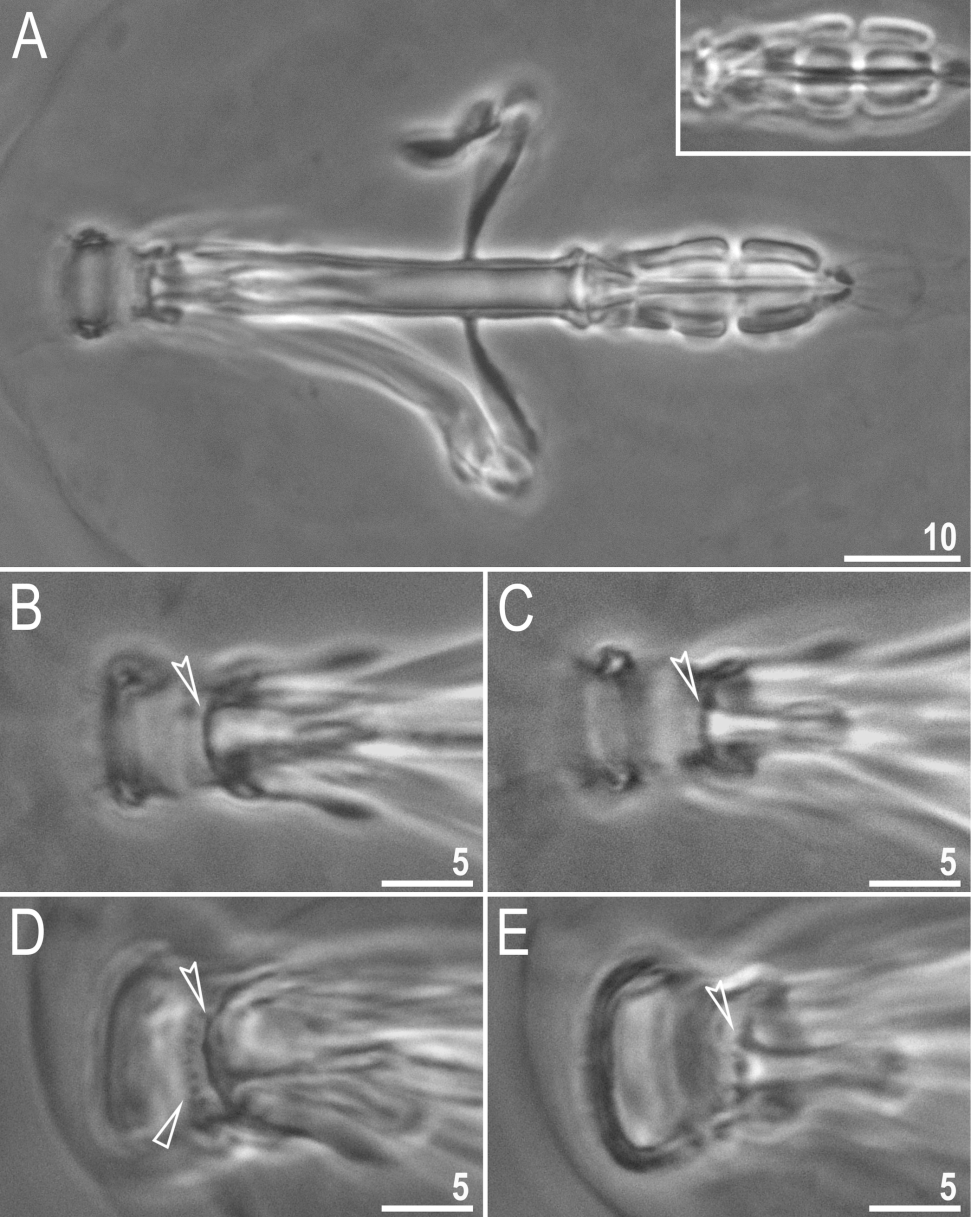
*Macrobiotus shonaicus* sp. nov.–buccal apparatus and the oral cavity armature seen in PCM (all paratypes). **A**–dorso-ventral projection with ventral teeth of the third band and ventral placoids, the upper insert shows dorsal placoids (a different individual); **B–C** oral cavity armature of the *maculatus* type (only the third band of teeth visible), dorsal and ventral view, respectively; and **D–E** oral cavity armature of the *patagonicus* type (both the second and the third band of teeth visible), dorsal and ventral view, respectively. Empty flat arrowhead indicates the second band of teeth in the oral cavity whereas empty indented arrowheads indicate third band of teeth. Fig A assembled from several photos. Scale bars in μm.

**Fig 4:**
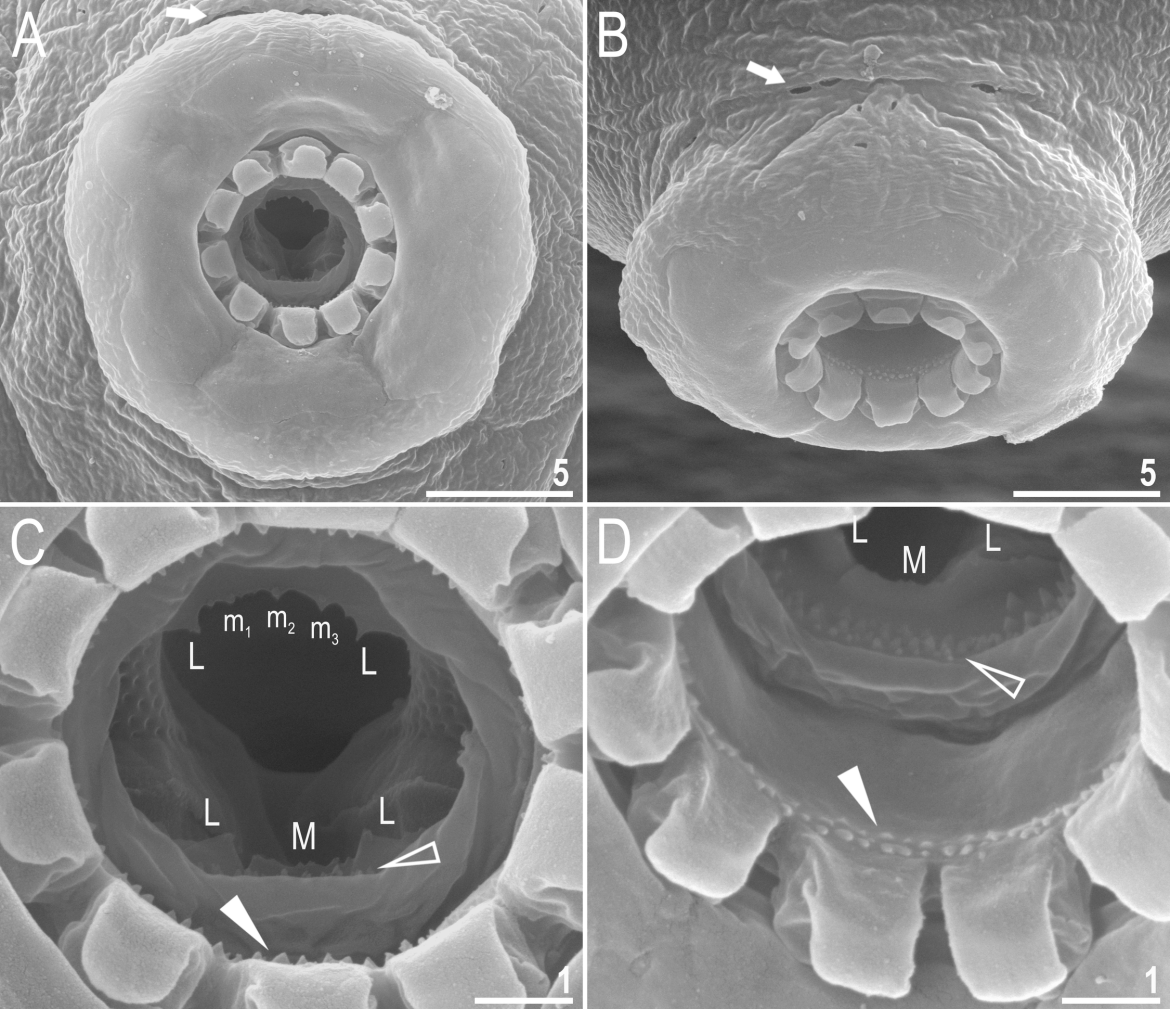
*Macrobiotus shonaicus* sp. nov.–mouth opening and the oral cavity armature seen in SEM (paratype). **A–B**–mouth opening with six peribuccal sensory lobes and ten lamellae; **C–D**–the oral cavity armature of a single paratype seen in SEM from different angles. Filled arrows indicate pores in the irregular ring of pores surrounding the peribuccal lobes, flat filled arrowheads indicate teeth of the first band, empty flat arrowheads indicate teeth of the second band, the peaks in the ridges of the third band that correspond with teeth of the third band in species with better developed oral cavity armatures are marked “m_1_-m_3_” (the dorso-median tooth comprises three small peaks visible as thickenings in PCM), “M” (ventro-median tooth) and “L” (lateral teeth). Scale bars in μm.

**Fig 5:**
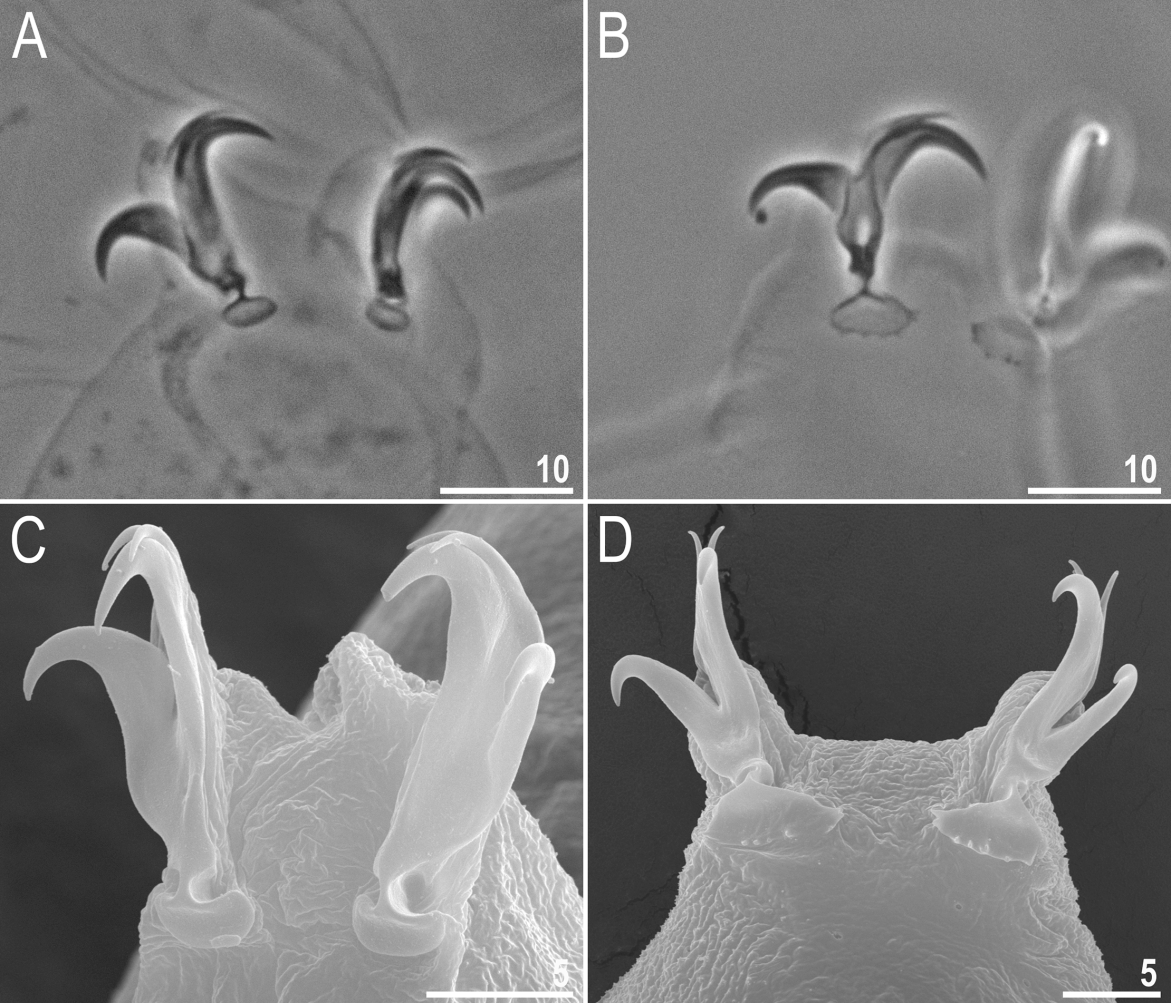
*Macrobiotus shonaicus* sp. nov.–claws (paratypes). **A–B**–claws I and IV seen in PCM, with smooth and slightly dentate lunules, respectively and **C–D**–claws I and IV seen in SEM, with smooth and slightly dentate lunules, respectively. Figs A and B assembled from several photos. Scale bars in μm.

**Fig 6:**
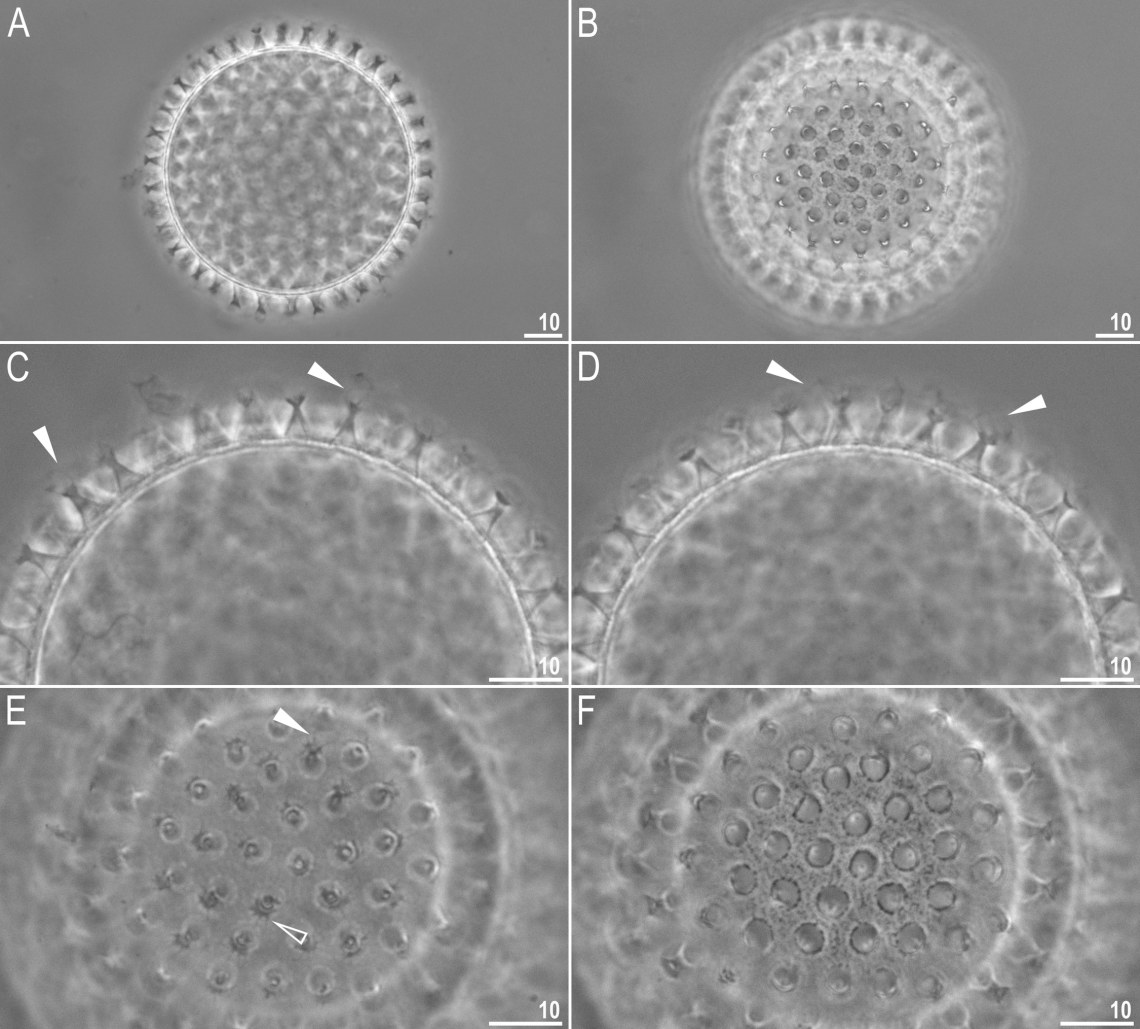
*Macrobiotus shonaicus* sp. nov.–egg seen in PCM. **A**–midsection under 400× magnification; **B**–surface under 400× magnification; **C–D–**midsection under 1000× magnification; **E–**surfaces of terminal discs under 1000× magnification; and **F**–surface of egg between processes under 1000× magnification (note fine dark dots between the processes). Flat filled arrowheads indicate thin flexible filaments whereas empty arrowhead indicate the indentation at the terminal disc edges. All photos show the details of a single egg. Scale bars in μm.

**Fig 7:**
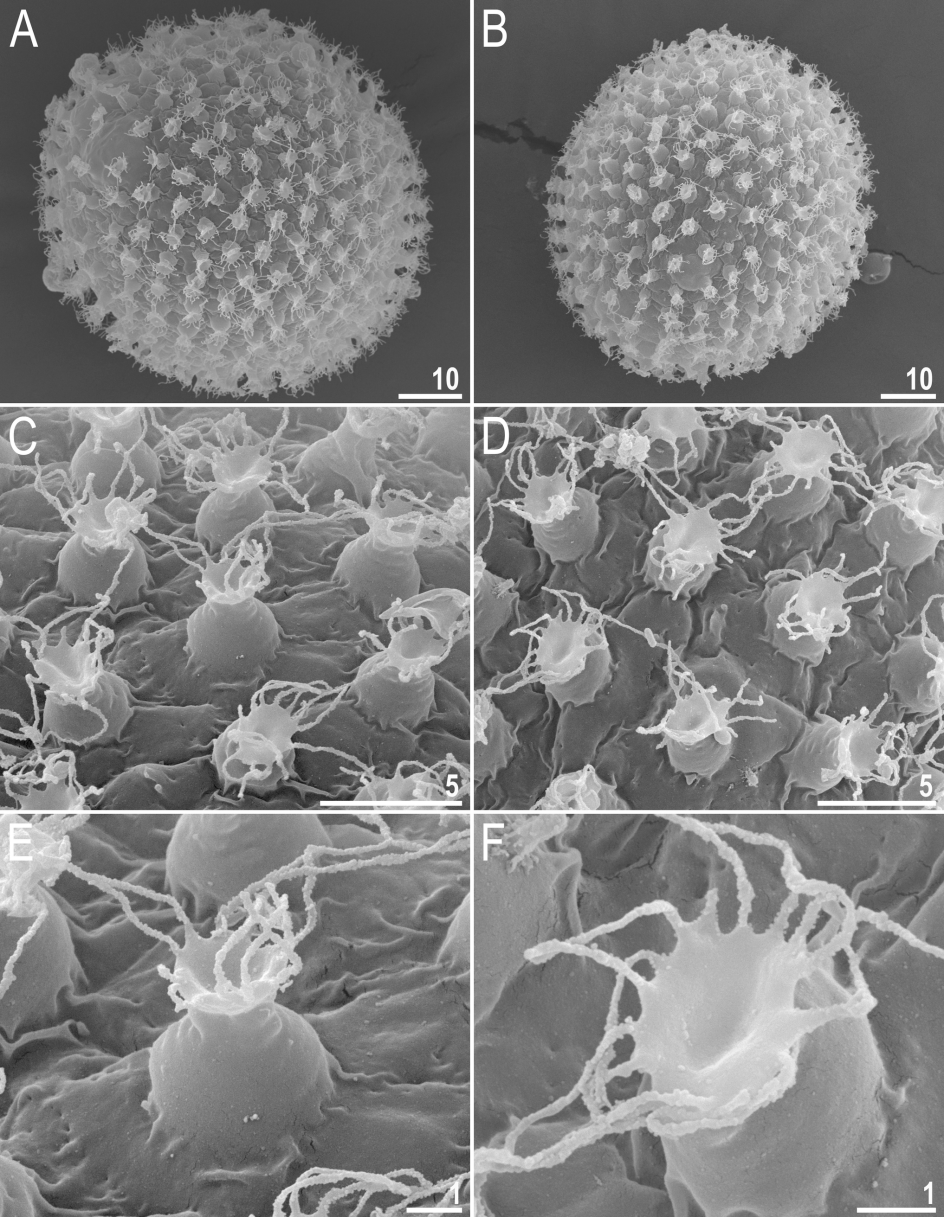
*Macrobiotus shonaicus* sp. nov.–egg chorion morphology seen in SEM. **A–B**–entire eggs with clearly visible flexible filaments on the egg processes; **C–D**–processes with filaments of various lengths and the surface between processes; and **E–F**–zoom on a single-egg process. Scale bars in μm.

***Macrobiotus shonaicus* sp. nov.** urn:lsid:zoobank.org:act:03337321-B020-4A81-B424-B1B4BC777DB1

(Tables 3 and 4, Figs 1–7)

**Table 3:** Measurements [in μm] and *pt* values of selected morphological structures of the holotype and paratypes of *Macrobiotus shonaicus* sp. nov. mounted in Hoyer’s medium (N–number of specimens/structures measured, RANGE refers to the smallest and the largest structure among all measured specimens; SD–standard deviation).

CHARACTER	N	RANGE						MEAN		SD		Holotype	
		μm			*pt*			μm	*pt*	μm	*pt*	μm	*pt*
Body length	30	318	–	743	*997*	–	*1658*	496	*1296*	97	*163*	494	*1235*
Buccopharyngeal tube							* *		* *		* *		
Buccal tube length	30	31.9	–	45.3		–	* *	38.0	–	3.3	–	40.0	–
Stylet support insertion point	30	23.0	–	32.5	*69*.*0*	–	*72*.*8*	27.3	*71*.*8*	2.4	*1*.*0*	28.7	*71*.*8*
Buccal tube external width	30	3.6	–	6.3	*10*.*3*	–	*15*.*8*	5.0	*13*.*2*	0.7	*1*.*2*	4.8	*12*.*0*
Buccal tube internal width	30	2.4	–	4.8	*7*.*0*	–	*11*.*7*	3.5	*9*.*3*	0.5	*1*.*1*	3.3	*8*.*3*
Ventral lamina length	24	16.0	–	25.5	*47*.*1*	–	*58*.*6*	21.0	*54*.*9*	2.2	*2*.*9*	21.6	*54*.*0*
Placoid lengths							* *		* *		* *		
Macroplacoid 1	29	7.8	–	14.9	*23*.*5*	–	*35*.*3*	10.7	*27*.*9*	1.9	*3*.*0*	10.6	*26*.*5*
Macroplacoid 2	29	4.5	–	10.0	*11*.*9*	–	*22*.*3*	6.5	*17*.*0*	1.4	*2*.*6*	7.3	*18*.*3*
Microplacoid	27	1.2	–	3.9	*3*.*8*	–	*9*.*9*	2.7	*7*.*1*	0.6	*1*.*3*	2.6	*6*.*5*
Macroplacoid row	29	13.4	–	25.4	*40*.*1*	–	*58*.*8*	18.5	*48*.*5*	3.2	*5*.*0*	19.3	*48*.*3*
Placoid row	27	15.1	–	29.5	*47*.*3*	–	*69*.*2*	21.9	*57*.*4*	3.5	*5*.*5*	22.1	*55*.*3*
Claw 1 lengths							* *		* *		* *		
External primary branch	28	10.1	–	15.8	*30*.*4*	–	*41*.*3*	13.4	*35*.*3*	1.4	*2*.*9*	13.5	*33*.*8*
External secondary branch	18	7.5	–	13.3	*23*.*5*	–	*35*.*2*	11.1	*28*.*8*	1.5	*3*.*2*	11.8	*29*.*5*
Internal primary branch	29	9.8	–	15.2	*29*.*2*	–	*39*.*1*	12.4	*32*.*6*	1.4	*2*.*5*	13.1	*32*.*8*
Internal secondary branch	19	7.9	–	12.3	*22*.*8*	–	*31*.*5*	10.0	*26*.*2*	1.2	*2*.*4*	10.0	*25*.*0*
Claw 2 lengths							* *		* *		* *		
External primary branch	29	11.2	–	17.7	*33*.*3*	–	*47*.*8*	14.9	*39*.*1*	1.6	*3*.*2*	14.8	*37*.*0*
External secondary branch	11	8.2	–	14.8	*25*.*7*	–	*32*.*7*	11.3	*29*.*6*	1.9	*2*.*3*	12.5	*31*.*3*
Internal primary branch	30	9.8	–	16.8	*29*.*6*	–	*45*.*9*	13.4	*35*.*2*	1.5	*3*.*6*	13.3	*33*.*3*
Internal secondary branch	22	7.4	–	12.5	*22*.*6*	–	*33*.*2*	10.6	*28*.*1*	1.1	*2*.*9*	10.6	*26*.*5*
Claw 3 lengths							* *		* *		* *		
External primary branch	27	11.2	–	18.8	*34*.*1*	–	*44*.*8*	14.9	*38*.*8*	1.8	*2*.*9*	15.3	*38*.*3*
External secondary branch	9	10.0	–	13.4	*26*.*1*	–	*33*.*0*	12.0	*30*.*6*	1.2	*2*.*6*	?	?
Internal primary branch	28	10.1	–	16.9	*30*.*5*	–	*40*.*3*	13.3	*34*.*8*	1.6	*2*.*8*	13.8	*34*.*5*
Internal secondary branch	17	8.3	–	14.0	*24*.*0*	–	*31*.*5*	10.8	*27*.*6*	1.4	*2*.*3*	11.0	*27*.*5*
Claw 4 lengths							* *		* *		* *		
Anterior primary branch	26	11.9	–	18.2	*34*.*2*	–	*48*.*4*	15.1	*39*.*8*	1.7	*3*.*8*	16.1	*40*.*3*
Anterior secondary branch	19	8.2	–	13.7	*25*.*3*	–	*35*.*0*	11.5	*29*.*7*	1.4	*2*.*9*	11.5	*28*.*8*
Posterior primary branch	26	13.0	–	20.5	*37*.*4*	–	*50*.*0*	16.3	*42*.*9*	1.8	*3*.*2*	16.7	*41*.*8*
Posterior secondary branch	14	9.4	–	16.3	*27*.*2*	–	*36*.*0*	12.2	*31*.*0*	1.8	*2*.*7*	?	?

**Table 4:** Measurements [in μm] of selected morphological egg structures of *Macrobiotus shonaicus* sp. nov. mounted in Hoyer’s medium (N–number of eggs/structures measured, RANGE refers to the smallest and the largest structure among all measured specimens; and SD–standard deviation).

CHARACTER	N	RANGE			MEAN	SD
Egg bare diameter	30	56.4	–	70.8	65.2	3.4
Egg full diameter	30	69.1	–	87.9	78.1	4.1
Process height	90	4.4	–	8.5	6.4	0.9
Process base width	90	3.1	–	6.5	4.5	0.7
Process base/height ratio	90	48%	–	109%	71%	10%
Terminal disc width	90	1.8	–	5.3	3.0	0.7
Inter-processes distance	90	1.5	–	4.2	2.4	0.5
Number of processes on the egg circumference	30	28	–	36	31.4	2.1

**Material examined:** 91 animals (including 3 simplex) and 49 eggs. Specimens mounted on microscope slides in Hoyer’s medium (75 animals + 41 eggs), fixed on SEM stubs (12+8), and processed for DNA sequencing (4+0).

### Description of the new species

#### Animals (measurements and statistics in Table 3)

Body white in juveniles and slightly yellowish in adults, transparent after fixation in Hoyer’s medium (Fig 1A). Eyes present in live animals (dissolved 33% of specimens mounted in Hoyer’s medium). Small round and oval pores (0.2–0.4 μm in diameter), invisible under PCM, but clearly visible under SEM, scattered randomly on the entire dorso-lateral cuticle (Fig 1B–1E), including the external and internal surface of all legs (Fig 2C, 2D and 2F, indented arrowheads). Cuticular granulation present on external surface of all legs (Fig 2A–2D). A cuticular bulge/fold resembling a pulvinus is present on the internal surface of all legs I–III (Fig 2E and 2F, flat filled arrowhead), whereas just above the claws, a faint cuticular fold is also present (Fig 2E and 2F, flat empty arrowhead). Both structures are visible only if the legs are fully extended and well oriented on the slide, especially the cuticular fold above the claws.

Mouth antero-ventral. Bucco-pharyngeal apparatus of the *Macrobiotus* type, with the ventral lamina and ten small peribuccal lamellae followed by six buccal sensory lobes (Figs 3A, 4A and 4B). An irregular ring of pores, visible only in SEM, is present around the mouth opening, immediately behind the peribuccal sensory lobes (Fig 4B, arrow). Under PCM, the oral cavity armature is of the *maculatus* type (only the third band of teeth visible under PCM) in smaller specimens and of the *patagonicus* type (only the second and third band of teeth visible under PCM) in larger specimens. However, in SEM the oral cavity is always composed of three bands of teeth, *i*.*e*., under PCM only the third band or second and third bands of teeth are visible (Fig 3B–3D), whereas all three bands are always detectable in SEM (Fig 4C and 4D). The first band of teeth is composed of numerous extremely small cones arranged in one to two rows situated anteriorly in the oral cavity, just behind the bases of the peribuccal lamellae (Fig 4C and 4D, flat filled arrowhead). The second band of teeth is situated between the ring fold and the third band of teeth and comprises 4–5 rows of small cones, slightly bigger than those of the first band (Fig 4C and 4D, empty flat arrowhead). Under PCM, only the bigger teeth of second band are visible, and they often appear as a single smudge rather than separate teeth (Fig 3D empty flat arrowhead). The teeth of the third band are located within the posterior portion of the oral cavity, between the second band of teeth and the buccal tube opening (Fig 4C and 4D). The third band of teeth is discontinuous and divided into the dorsal and the ventral portions. Under PCM, the dorsal teeth form a single transversal ridge with poorly visible thickenings, whereas the ventral teeth appear as two separate lateral transversal ridges between which a roundish median tooth is sometimes visible (Fig 3B–3E). However, in SEM, both dorsal and ventral teeth form two single ridges (Fig 4C and 4D), although with peaks. The dorsal ridge has two larger lateral peaks and several smaller median peaks and indentations (Fig 4C), which correspond to thickenings sometimes visible in this band under PCM (Fig 3B and 3D). The ventral ridge has two evident peaks corresponding with two lateral teeth, between which the ventral ridge is reduced/indented and only slightly serrated (Fig 4C). This median portion can be seen as roundish median tooth under PCM, especially in larger specimens (Fig 3E). Pharyngeal bulb spherical with triangular apophyses, two rod-shaped macroplacoids and a triangular small microplacoid (Fig 3A). Macroplacoid length sequence 2<1. The first and the second macroplacoid have a constriction, central and subterminal, respectively (Fig 3A, upper insert).

Claws small and slender, of the *hufelandi* type (Fig 5A–5D). Primary branches with distinct accessory points, a long common tract, and with an evident stalk connecting the claw to the lunula (Fig 5A–5D). Lunulae on legs I–III smooth (Fig 5A and 5C), whereas on legs IV, the lunules are sparsely dentate (Fig 5B and 5D). Cuticular bars under claws absent.

The population type is dioecious. Males were discovered using aceto-orcein staining, which revealed testicles filled with spermatozoa. The dioecism (gonochory) was also confirmed experimentally by isolating virgin individuals that have never reproduced, suggesting that females cannot reproduce parthenogenetically. No morphological secondary sexual dimorphism, such as gibbosities on legs IV in males, was identified.

#### Eggs (measurements and statistics in Table 4)

Laid freely, white/light yellow, spherical or slightly oval (Figs 6A, 6B, 7A and 7B). The surface between processes of the *persimilis* type, *i*.*e*., chorion surface solid, without pores or reticulum, covered by irregularly shaped and sized convex cushion-like platforms (Fig 7C and 7D). Superimposed on these platforms, and at their margins, are occasional vein-like folds and extensions (Fig 7E). The complex and intricate intersection of these platforms (Fig 7D and 7E) forms the dark dots seen under PCM (Fig 6F). Processes are in the shape of inverted goblets with slightly concave conical trunks and well-defined terminal discs (Figs 6B, 6D and 7C–7F). Terminal discs are indented (cog-shaped) with a concave central area and with 10–15 small irregular teeth (Figs 6E and 7C–7F). Almost all teeth on the terminal disc are elongated into thin flexible filaments, less than 0.2 μm in diameter and 2–5 μm in length (Figs 6C–6E, flat filled arrowhead and 7C–7F). The filaments are hair-like under PCM, but under SEM, they are covered with fine granulation (Fig 7E and 7F), so they probably enhance the adhesive function of egg processes. Under PCM the filaments are sometimes barely visible or even invisible. The most likely explanation for this is that the filaments are very fragile and easily broken; thus, they are not present on some eggs due to mechanical damage. The filaments are very thin, so they could be overlooked and/or misinterpreted as debris attached to eggs. Thus, extreme care must be taken when examining the eggs to avoid incorrect conclusions.

#### DNA sequences

We obtained very good quality sequences for all four molecular markers from all four analyzed specimens (paragenophores). The 18S rRNA and 28S rRNA sequences were represented by single private haplotypes, whereas the ITS-2 and COI were represented by two private haplotypes differing in two (p-distance: 0.9%) and eight variable sites (p-distance: 1.0%), respectively:

The **18S rRNA** sequence (GenBank: MG757132), 1038 bp long:

TAGATCGTAATCTTACACGGATAACTGTGGTAATTCTAGAGCTAATACGTGCAACCAGCTCGTTCCCTTGTGGAGCGAGCGCAGTTATTAGAACAAGACCAATCCGGCCTTCGGGTCGGTACAATTGGTGACTCTGAATAACCGAAGCGGAGCGCATGGTCTCGTACCGGCGCCAGATCTTTCAAGTGTCTGACTTATCAGCTTGTTGTTAGGTTATGTTCCTAACAAGGCTTCAACGGGTAACGGGGTATCAGGGTCCGATACCGGAGAGGGAGCCTGAGAAACGGCTACCACATCCAAGGAAGGCAGCAGGCGCGCAAATTACCCACTCCTAGCACAGGGAGGTAGTGACGAAAAATAACGATGCGAGGGCTAATAGCTTCTCGTAATCGGAATGGGTACACTTTAAATCCTTTAACGAGGATCTATTGGAGGGCAAGTCTGGTGCCAGCAGCCGCGGTAATTCCAGCTCCAATAGCGTATATTAAAGTTGCTGCGGTTAAAAAGCTCGTAGTTGGATCTGGGCTTCTGAATGGATGGTTCACTTTACGGTGTAACTGTTCGTTTGGTGCCACAAGCCGGCCATGTCTTGCATGCCCTTTACTGGGTGTGCTTGGCGACCGGAACGTTTACTTTGAAAAAATTAGAGTGCTCAAAGCAGGCGTATGGCCTTGCATAATGGTGCATGGAATAATGGAATAGGACCTCGGTTCTATTTTGTTGGTTTTCGGAACTCGAGGTAATGATTAAGAGGAACAGACGGGGGCATTCGTATTGCGGCGTTAGAGGTGAAATTCTTGGATCGTCGCAAGACGAACTACTGCGAAAGCATTTGCCAAGAATGTTTTCATTAATCAAGAACGAAAGTTAGAGGTTCGAAGGCGATCAGATACCGCCCTAGTTCTAACCATAAACGATGCCAACCAGCGATCCGTCGGTGTTTTTTTTATGACTCGACGGGCAGCTTTCCGGGAAACCAAAGTGCTTAGGTTCCGGGGGAAGTATGGTTGCAAAGCTGAAACTTAAAGGAATGACGAA.

The **28S rRNA** sequence (GenBank: MG757133), 786 bp long:

TACTAAGCGGAGGAAAAGAAACCAACGGGGATGCCGAGAGTAACTGCGAGTGAAATCGGCCAAGCCCAGCGCCGAATCCTGTTGcTGGTGACGGTGACAGGAACTGTGGCGTGAAGAACGTCCTTACCGGTACGGTTTGCGTGCGTAAGTTCTCCTGAGTGAGGCTCCATTCCAAGGAGGGTGCAAGACCCGTATCGCGTGCAACCGGTATCGGTGTAAGATGTTCGGAGAGTCGCCTTGTTTGTGAGTACAAGGTGAAGTCGGTGGTAAACTCCATCGAAGGCTAAATATGACCACGAGTCCGATAGCGAACAAGTACCGTGAGGGAAAATTGAAAAGCACTTTGAAGAGAGAGCGAAACAGTGCGTGAAACCGCTCAGAGGCAAGCAAATGGGGCCTCGAAGGCAAGGCAGCGAATTCAGCTGGTGGTCTGCGTGGCTGGCTGGTTAAGTGATCTTAACGACTCTTGCCGGTCATGTCTAGCGTGGGTGCCAGTGCACTTTYGTTGCTTGTACGCCACCGCCGTTGAGTGGGCATCCGTCGGGTAGGCAATACGAAGCCTTAAGCCTTTACGGGCCTAGGTGCTTGTAGTCTGCTTTGTACGCGTTTGCACTTCAACCGGTCATGTTTGCATGTGTCAGCATTTGCGTTGGATTGGCTCGCTCTGCCGTTTGTCTGGGAAGACGAGCTTGCTCGGCTCCTGGGCATGTATGGTAGAATCGTGTCGGTTTTCAACGTGGGCACATTGTTAATTCGGTGGCGAGTAGATGGCTGCCCATTTAACCC.

The **ITS-2** *haplotype 1* sequence (GenBank: MG757134), 337 bp long (variable sites bolded):

ACGCACATTGCGGCTTCGGGTTAACTGAAGCCATGCCTGGTTGAGGGTCAGTTGAAGAAAAAAATCGTAATCGCGCATTGATTACGGATTGTCTGGTTAATGGCTTCGGTCGTTTCCAGATGAAGTTGAGACCAGATGTGTGCGCTCGTTTGACTGGTGGCAAAACGCTTTGCCGAGTTGGAGCATCCGGCTTTCCTAGCCGTGCGCCGCAGCTGCACGATGGTTAGGTTGGCCAACCAACTGCGATTGATGGCAAAGTTACCGGTTC**G**AAAGTGCGCAA**A**GCAATAGGCACATCTGTGAGCCAGAAAAGTTTGTGTTGGTTGCAGTGTTGACCGAC.

The **ITS-2** *haplotype 2* sequence (GenBank: MG757135), 337 bp long (variable sites bolded):

ACGCACATTGCGGCTTCGGGTTAACTGAAGCCATGCCTGGTTGAGGGTCAGTTGAAGAAAAAAATCGTAATCGCGCATTGATTACGGATTGTCTGGTTAATGGCTTCGGTCGTTTCCAGATGAAGTTGAGACCAGATGTGTGCGCTCGTTTGACTGGTGGCAAAACGCTTTGCCGAGTTGGAGCATCCGGCTTTCCTAGCCGTGCGCCGCAGCTGCACGATGGTTAGGTTGGCCAACCAACTGCGATTGATGGCAAAGTTACCGGTTC**A**AAAGTGCGCAA**G**GCAATAGGCACATCTGTGAGCCAGAAAAGTTTGTGTTGGTTGCAGTGTTGACCGAC.

The **COI** sequence *haplotype 1* (GenBank: MG757136), 658 bp long (variable sites bolded):

**A**AC**A**TTGTACTTTATATTCGGACTTTG**G**ACGGCTTGTG**C**CGGGACATCTTTAAGCTTCTTAATTCGAACAGAATTAAGACAACCTGGTCTTTTATTTTCAGATGAACAGCTGTACAATGTAATTGTTACCAGTCACGCATTTGTTATAATTTTCTTCTTTGTGATACCAGTTTTAATCGGAGGATTCGGAAATTGACTTGTACCTCTAATAATTAGAGCCCCCGATATGGC**A**TTTCCTCGAATAAACAATCTTAGATTTTGAATGCTTCCTCCCTCATTTTTTTTAATTACAATTAG**A**TCAATAGCAGAACAAGGGGCCGGAACAGGATGAACTGTATACCC**C**CCCCTATCCCATTTTTTTGCTCACAGTGGACCAAGTGTAGACTTAACTATTTTTTCACTTCACGTAGCAGGAATTTCTTCCATTTTAGGAGCTATTAATTTCATTTCTACAATTATAAATATGCGAGCTCCCCATTTAAGATTAGATAAAATACCCTTATTTGTTTGATCTGTTTTACTAACAGCTATCCTACTA**C**TACTAGCTTTACCTGTTCTGGCGGGAGGAATTACAATACTTCTCTTAGACCGAAACTTCAATACATCTTTCTTCGATCCTGCAGGGGGAGGGGACCCAATCCTCTATCAACACTTATTT.

The **COI** sequence *haplotype 2* (GenBank: MG757137), 658 bp long (variable sites bolded):

**G**AC**G**TTGTACTTTATATTCGGACTTTG**A**ACGGCTTGTG**T**CGGGACATCTTTAAGCTTCTTAATTCGAACAGAATTAAGACAACCTGGTCTTTTATTTTCAGATGAACAGCTGTACAATGTAATTGTTACCAGTCACGCATTTGTTATAATTTTCTTCTTTGTGATACCAGTTTTAATCGGAGGATTCGGAAATTGACTTGTACCTCTAATAATTAGAGCCCCCGATATGGC**G**TTTCCTCGAATAAACAATCTTAGATTTTGAATGCTTCCTCCCTCATTTTTTTTAATTACAATTAG**G**TCAATAGCAGAACAAGGGGCCGGAACAGGATGAACTGTATACCC**T**CCCCTATCCCATTTTTTTGCTCACAGTGGACCAAGTGTAGACTTAACTATTTTTTCACTTCACGTAGCAGGAATTTCTTCCATTTTAGGAGCTATTAATTTCATTTCTACAATTATAAATATGCGAGCTCCCCATTTAAGATTAGATAAAATACCCTTATTTGTTTGATCTGTTTTACTAACAGCTATCCTACTA**T**TACTAGCTTTACCTGTTCTGGCGGGAGGAATTACAATACTTCTCTTAGACCGAAACTTCAATACATCTTTCTTCGATCCTGCAGGGGGAGGGGACCCAATCCTCTATCAACACTTATTT.

**Type locality:** 38°44’24”N, 139°48’26”E; 13 m asl: Japan, Tsuruoka-City, Otsuka-machi, car park; *Bryum argenteum* moss growing on concrete; coll. 05.2016.

**Etymology:** The name ‘*shonaicus*’ refers to Shōnai (庄内), the region in Japan where the new species was collected.

**Type depositories:** Holotype: **slide JP.002.05**, 57 paratypes (slides: JP.002/*, where the asterisk can be substituted by any of the following numbers 04, 06–10, 17–22) and 34 eggs (slides: JP.002/*: 01–03) are deposited at the Department of Entomology, Institute of Zoology and Biomedical Research, Jagiellonian University, Gronostajowa 9, 30–387, Kraków, Poland and 17 paratypes (slides: JP.002/*: 11–13, 15, 16) and 7 eggs (slide JP.002.14) are deposited at the Institute for Advanced Biosciences, Keio University, Tsuruoka, Japan.

#### Molecular phylogeny

**Fig 8:**
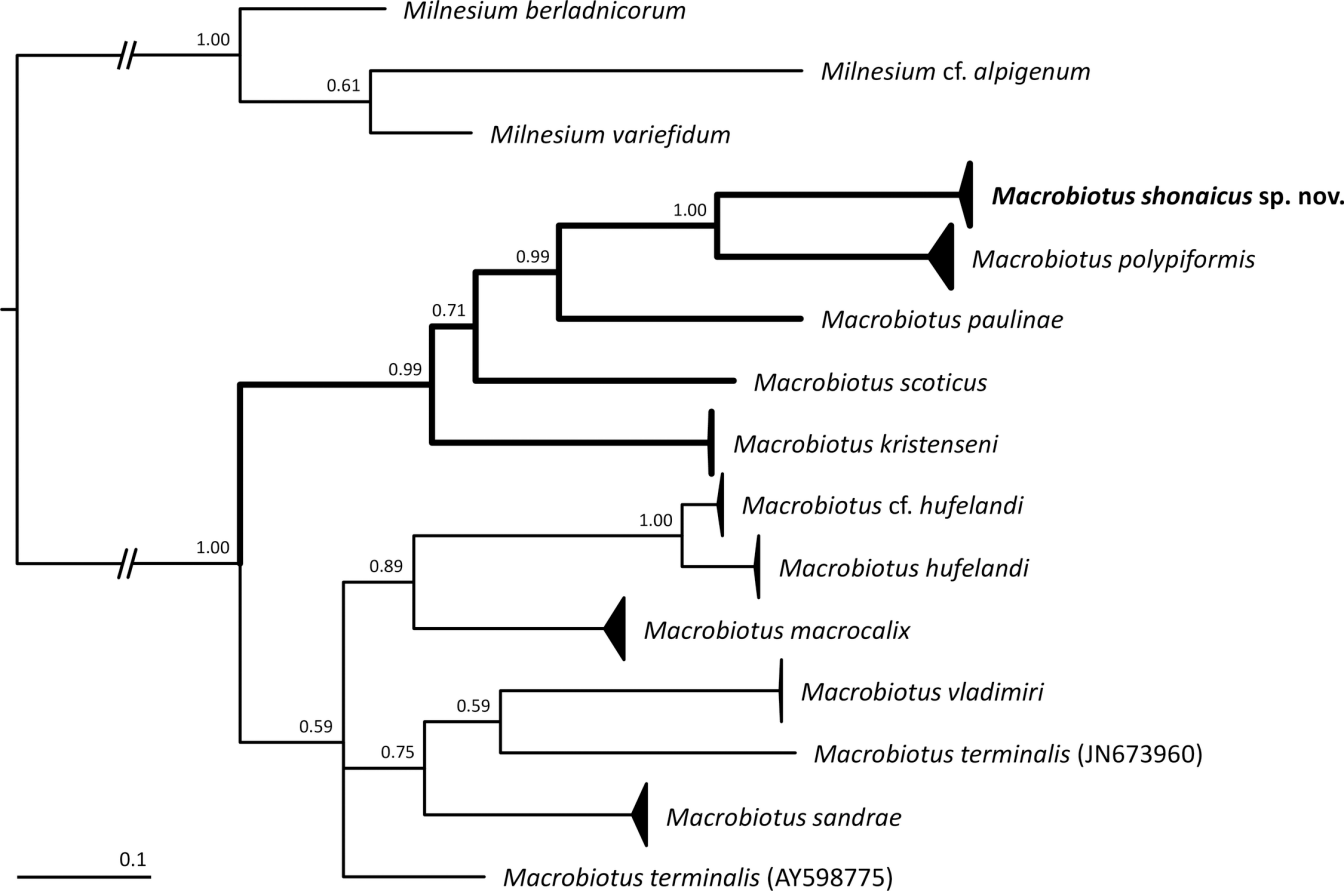
The Bayesian Inference (BI) phylogeny constructed from COI sequences of the *Macrobiotus hufelandi* group species. Numbers at nodes indicate Bayesian posterior probability. The *hufelandi* species clade with modified egg processes is indicated by thicker branches. Please see Table 2 for details on species sequences used in the analysis. Scale bar represents substitutions per position.

The phylogenetic analysis of the available COI sequences for the *M*. *hufelandi* group unequivocally showed that *M*. *shonaicus* **sp. nov.** indeed belongs to the species complex (Fig 8). However, more interestingly, the analysis also revealed that the new species clusters within a single clade with the two other known species of the group that exhibit flexible filaments on terminal discs of egg processes, *i*.*e*., *M*. *polypiformis* and *M*. *paulinae* (Fig 8). Moreover, the clade clusters with two further species with atypical egg processes for which COI sequences are available, *i*.*e*., *M*. *scoticus* and *M*. *kristenseni*. In these two species, egg processes are strongly modified and do not resemble the typical mushroom-shaped processes found most commonly within the *hufelandi* complex [21] (Fig 8). The remaining sequenced *hufelandi* group taxa, all exhibiting typical inverted goblet-shaped egg processes (*i*.*e*., *M*. *hufelandi*, *M*. cf. *hufelandi*, *M*. *macrocalix*, *M*. *vladimiri*, *M*. *terminalis*, and *M*. *sandrae*), are grouped within a sister clade. The clades are well supported using Bayesian analysis (Fig 8) but weakly supported using the Maximum Likelihood method. Supports of each node on the ML tree were very small (most of the clades have the bootstrap support value <50, indicating a widespread polytomy). Nevertheless, in both analyses, the five species with modified egg processes always grouped together, suggesting their closer affinity compared to any other *hufelandi* species included in the analysis, even though they come from four different continents (South America, Africa, Europe and Asia).

## Discussion

### Phenotypic differential diagnosis

By the presence of the *maculatus* or *patagonicus* OCA type and eggs of the *persimilis* type, *M*. *shonaicus* **sp. nov.** is most similar to the following species of the *hufelandi* group: *M*. *anemone*, *M*. *naskreckii*, and *M*. *patagonicus*. However, the new species can be easily distinguished from these species by a having cuticular bulge/fold (resembling a pulvinus) on the internal surface of legs I–III and by thin flexible filaments at the terminal disc of egg process. Moreover, *M*. *shonaicus* **sp. nov.** differs specifically from the following:

- *M*. *anemone*, reported from its *terra typica* (Louisiana, USA) and several localities in southeastern USA [23, 71, 72, 73] by: the presence of eyes (eyes absent in *M*. *anemone*), the presence of granulation on all legs (the granulation is absent in *M*. *anemone*), the presence of sparsely dentate lunulae on legs IV (all lunules smooth in *M*. *anemone*), a lower *pt* of the stylet support insertion point (*69*.*0–72*.*8* in the new species *vs*. *78*.*0–79*.*2* in *M*. *anemone*), a higher *pt* of the internal primary branch II (*30*.*5–40*.*3* in the new species *vs*. *25*.*7–27*.*7* in *M*. *anemone*), a higher *pt* of the internal secondary branch III (*24*.*0–31*.*5* in the new species *vs*. *20*.*3–23*.*2* in *M*. *anemone*), the presence of evidently shorter teeth on the terminal discs of eggs processes, the presence of irregular thickenings on the egg surface between the processes, seen in PCM as dark dots in the new species *vs*. a smooth egg surface under PCM in *M*. *anemone*, the smaller diameters of eggs without processes (56.4–70.8 μm in the new species *vs*. 75.0–94.3 μm in *M*. *anemone*), and by a smaller inter-process distance (1.5–4.2 μm in the new species *vs*. 4.3–7.3 μm in *M*. *anemone*)
- *M*. *naskreckii*, reported only from the *locus typicus* (Mozambique) [29, 74] by: smaller cuticular pores (not visible under PCM in the new species *vs*. cuticular pores clearly visible under PCM in *M*. *naskreckii*), the presence of a subterminal constriction in the second macroplacoid (the subterminal constriction is absent in *M*. *naskreckii*), a lower *pt* of the stylet support insertion point (*69*.*0–72*.*8* in the new species *vs*. *74*.*1–77*.*8* in *M*. *naskreckii*), a higher *pt* of the macroplacoid 1 length (*23*.*5–35*.*3* in the new species *vs*. *19*.*0–22*.*8* in *M*. *naskreckii*), a different egg surface morphology between the processes (irregular thickenings visible in PCM as dark dots and irregularly shaped and sized convex cushion-like platforms, covered occasional vein-like folds and extensions under SEM in the new species *vs*. shallow depressions forming pseudoareoles in *M*. *naskreckii*), and a different morphology of egg processes (inverted goblet shape processes with indented terminal discs with a concave central area and 10–15 small irregular teeth in the new species *vs*. conical processes with extremely reduced terminal discs shaped as a crown of short finger-like appendages in *M*. *naskreckii*).
- *M*. *patagonicus*, reported from *locus typicus* (Argentina) and several other localities in Argentina and Chile [30, 75, 76] by: a considerably constricted first macroplacoid (a fine constriction present only in some paratypes of *M*. *patagonicus*), sparsely dentate lunules IV (smooth in *M*. *patagonicus*), a lower mean *pt* for the stylet support insertion point (*71*.*8±1*.*0* in the new species vs. *78*.*8±2*.*3* in *M*. *patagonicus*), a different egg surface morphology between the processes (irregular thickenings visible in PCM as dark dots and irregularly shaped and sized convex cushion-like platforms, covered occasional vein-like folds and extensions under SEM in the new species *vs*. smooth egg surface under PCM in *M*. *patagonicus*), and a smaller bare egg diameter (56.4–70.8 μm [mean: 65.2*±*3.4 μm] in the new species *vs*. 70.0–115.0 μm [mean: 98.3*±*11.7 μm] in *M*. *patagonicus*).

The presence of flexible filaments at the terminal disc of egg processes *M*. *shonaicus* **sp. nov.** is similar to two species of the *hufelandi* subgroup: *M*. *paulinae* and *M*. *polypiformis*. However, the new species can easily be distinguished from both species by having a solid egg surface between processes (*persimilis* egg type) instead of the surface covered by the reticulum (*hufelandi* egg type), a cuticular fold on the internal surface of legs I–III, smaller pores (below PCM resolution in the new species *vs*. identifiable under PCM in *M*. *paulinae* and *M*. *polypiformis*), a different morphology of the ventral teeth in the third band under PCM (two separate lateral transversal ridges with a roundish median tooth between them in the new species *vs*. a single ventral thin transverse ridge in *M*. *paulinae* and *M*. *polypiformis*), and by the presence of a subterminal constriction in the second macroplacoid (the constriction is absent in *M*. *paulinae* and *M*. *polypiformis*). Moreover, the new species differs specifically from the following:

- *M*. *paulinae*, reported only from the type locality (Kenya) [11, 74] by: the absence of body granulation (seven dorso-lateral patches of sparse and minute granulation arranged symmetrically on both sides of the body in *M*. *paulinae*), the presence of only a single granulated patch on the external surface of legs I–III (two distinct patches: a small area of fine and dense granulation just above the claws and a larger area of more robust and sparse granulation located in the middle of each leg in *M*. *paulinae*), and a smaller diameter of the flexible filaments on the egg processes (less than 0.2 μm in the new species *vs*. approximately 0.5 μm in *M*. *paulinae*).
- *M*. *polypiformis*, reported only from the type locality (Ecuador) [12] by the longer first macroplacoid (7.8–14.9 μm [*pt = 23*.*5*–*35*.*3*] in the new species *vs*. 5.2–6.8 μm [*pt = 19*.*2*–*23*.*5*] in *M*. *polypiformis*), a slightly longer second macroplacoid (4.5–10.0 μm [*pt = 11*.*9*–*22*.*3*] in the new species *vs*. 2.8–4.1 μm [*pt = 11*.*4*–*14*.*5*] in *M*. *polypiformis*), a longer macroplacoid row (13.4–25.4 μm [*pt = 40*.*1*–*58*.*8*] in the new species *vs*. 9.0–11.8 μm [*pt = 34*.*3*–*39*.*9*] in *M*. *polypiformis*), a slightly longer placoid row (15.1–29.5 μm [*pt = 47*.*3*–*69*.*2*] in the new species *vs*. 11.1–14.5 μm [*pt = 41*.*4*–*49*.*0*] in *M*. *polypiformis*), and the smaller number of processes on the egg circumference (28–36 in the new species *vs*. 19–23 in *M*. *polypiformis*) and by thinner flexible filaments at terminal disc edges of egg processes (diameter of filaments less than 0.2 μm in the new species *vs*. almost always more than 0.2 μm in *M*. *polypiformis*; measurements based on SEM photomicrographs).

### Genotypic differential diagnosis

The ranges of uncorrected genetic p-distances between the new species and species of the *Macrobiotus hufelandi* complex, for which sequences are available from GenBank, are as follows (from the most to the least conservative):

- **18S rRNA**: 0.3–3.3% (2.0% on average), with the most similar being an undetermined *M*. *hufelandi* group species from Italy (HQ604971) and the least similar being *M*. *polonicus* from Poland (HM187580);
- **28S rRNA**: 5.0–9.8% (8.4% on average), with the most similar being *M*. *paulinae* from Kenya (KT935501) and the least similar being an undetermined *M*. *hufelandi* group species from Spain (FJ435751, FJ435754–5);
- **ITS-2**: 11.2–29.7% (23.3% on average), with the most similar being *M*. *sapiens* from Croatia (GQ403680) and the least similar being *M*. *polonicus* from Poland (HM150647).
- **COI**: 19.6–25.9% (23.4% on average), with the most similar being *M*. *polypiformis* from Ecuador (KX810011) and the least similar being *M*. cf. *hufelandi* from Switzerland (HQ876589–94) and Italy (HQ876596);

### Phylogenetic position within the *hufelandi* group

According to our phylogenetic analysis, *M*. *shonaicus* **sp. nov.** forms a distinct clade together with other species with modified egg processes, *i*.*e*., with *M*. *paulinae*, *M*. *polypiformis*, *M*. *scoticus* and *M*. *kristenseni*. Thus, it may be hypothesized that the ancestor of the mentioned species with atypical egg processes might have exhibited a mutation allowing derivations from the inverted goblet-like shape of egg processes. Considering fewer morphological differences between animals of these species compared to those observed in egg morphology, our results show that chorion can evolve faster than animal anatomy, which is consistent with previous studies [16, 27, 36]. This phenomenon makes egg ornamentation particularly useful for the delineation of closely related species.

Even though the BI tree was much better supported than the ML tree, the number of available COI sequences for the *hufelandi* group species remains rather small. Therefore, a greater effort should be made to increase the sample size to obtain more reliable (and preferably multilocus) phylogenies that would allow testing to determine whether the two lineages revealed in our study indeed represent biological entities.

## Conclusions

Thanks to the integrative approach of combining morphological, morphometric and molecular analysis, *Macrobiotus shonaicus* **sp. nov.** has been unambiguously delimited from its congeners as a new species. The most characteristic traits of the new species are cuticular folds on the internal surfaces of all legs I–III and eggs with solid surface between processes on which teeth of terminal discs are elongated to thin flexible filaments. Moreover, our phylogenetic analysis showed that species of the *Macrobiotus hufelandi* complex that exhibit modified egg processes are closely related and form a distinct clade. This is the first original description of the *hufelandi* group species from Japan, and now, the number of tardigrade species known from this country has increased to 168.

## Supporting information

S1 File Measurements. Raw measurements of animals and eggs of *Macrobiotus shonaicus* sp. nov.
(XLSX)

S2 File Genetic distances. Matrices with calculated uncorrected p-genetic distances between *Macrobiotus shonaicus* sp. nov and other species of *Macrobiotus hufelandi* complex.
(XLSX)
